# Survival characteristics of Wilms Tumor, a reference developed from a longitudinal cohort study

**DOI:** 10.1186/s13052-024-01698-7

**Published:** 2024-08-06

**Authors:** Anas Elgenidy, Ahmed M. Afifi, Eman F. Gad, Hoda Atef Abdelsattar Ibrahim, Ubaid khan, Omar Alomari, Huzaifa A. Cheema, Mohammad Ebad-Ur-Rehman, Aya Sherif, Mohammad Alzu’bi, Mohamed Abd-Elfattah, Ahmad Roshdy Ahmad, Amira Elhoufey, Amira M. Osman, Mohamed Ezzat, Ahmed E. Hammour, Hamad Ghaleb Dailah, Doaa Ali Gamal, Khaled Saad

**Affiliations:** 1 Faculty of Medicine, Cairo University, Cairo, Egypt; 2 Department of Surgery, University of Toledo Medical Center, Toledo, OH USA; 3 Department of Pediatrics, Faculty of Medicine, Assiut University, Assiut, Egypt; 4 Pediatric Clinical Nutrition Unit, Department of Pediatrics, Faculty of Medicine, Cairo University, Cairo, Egypt; 5 Department of Pediatrics, Cairo University, Cairo, Egypt; 6 Department of Medicine, King Edward Medical University, Lahore, Pakistan; 7 Department of Medicine, Hamidiye International Faculty of Medicine, University of Health Sciences, Istanbul, Turkey; 8 Department of Medicine, Rawalpindi Medical University, Rawalpindi, Pakistan; 9 Department of Medicine, Faculty of Medicine, Beni Suef University, Beni Suef, Egypt; 10 Department of Medicine, Faculty of Medicine, Hashemite University, Zarqa, Jordan; 11 Department of Pediatrics, College of Medicine, Jouf University, Sakaka, Saudi Arabia; 12 Department of Community Health Nursing, Alddrab University College, Jazan University, 45142 Jazan, Saudi Arabia; 13 Department of Pediatric Oncology, South Egypt Cancer Institute, Assiut University, Assiut, Egypt; 14 Department of Pediatrics, Faculty of Medicine, Al Azhar University, Cairo, Egypt; 15 Research and Scientific Studies Unit, College of Nursing, Jazan University, Jazan, Saudi Arabia; 16 Department of Clinical Oncology Faculty of Medicine, Assiut University, Assiut, Egypt

## Abstract

**Background:**

Wilms tumor (WT) survival has been affected by the evolution in clinical and biological prognostic factors. Significant differences in survival rates indicate the need for further efforts to reduce these disparities. This study aims to evaluate the clinicopathological data impact on survival among patients after Wilm's diagnosis.

**Methods:**

The study utilized the SEERStat Database to identify Wilms tumor patients, applying SEERStat software version 8.3.9.2 for data extraction. Selection criteria involved specific codes based on the International Classification of Diseases for Oncology (ICDO-3), excluding cases with unknown SEER stage, incomplete survival data, unknown size, or lymph node status. Statistical analyses, including Kaplan–Meier estimates and Cox regression models, were conducted using R software version 3.5. Standardized mortality ratios (SMR) were computed with SEER*Stat software, and relative and conditional survival analyses were performed to evaluate long-term survival outcomes.

**Results:**

Of 2273 patients diagnosed with Wilms tumor, (1219 patients, 53.6% were females with an average age group of 3–8 years (50.2%). The overall mean survival after five years of diagnosis was 93.6% (2.6–94.7), and the overall mean survival rate was 92.5% (91.3–93.8) after ten years of diagnosis. Renal cancers were identified as the leading cause of death (77.3%), followed by nonrenal cancers (11%) and noncancer causes (11%). Additionally, robust relative survival rates of 98.10%, 92.80%, and 91.3% at one, five, and ten years, respectively, were observed, with corresponding five-year conditional survival rates indicating an increasing likelihood of survival with each additional year post-diagnosis. Univariate Cox regression identified significant prognostic factors: superior CSS for patients below 3 years (cHR 0.48) and poorer CSS for those older than 15 years (cHR 2.72), distant spread (cHR 10.24), regional spread (cHR 3.09), and unknown stage (cHR 4.97). In the multivariate model, age was not a significant predictor, but distant spread (aHR 9.22), regional spread (aHR 2.84), and unknown stage (aHR 4.98) were associated with worse CSS compared to localized tumors.

**Conclusion:**

This study delving into WT survival dynamics reveals a multifaceted landscape influenced by clinicopathological variables. This comprehensive understanding emphasizes the imperative for ongoing research and personalized interventions to refine survival rates and address nuanced challenges across age, stage, and tumor spread in WT patients.

## Impact Statement

What is already known regards this study?

Prognostic factors for survival in Wilms tumor are previously studied in different reports.

## What does this study add?

This study may be one of the few studies that collectively investigate the impact of multiple risk factors together on the outcome and trace how these factors ultimately can affect the prognosis. Furthermore, this report enrolled a large sample size allowing for generalized validity of the study. In addition, the longer period of this study investigating the prognosis at 1, 5, and 10 years of diagnosis adds a significant value. Moreover, this study may be a guide for risk stratification of children with Wilms tumor allowing early intervention.

## Introduction

Wilms tumor (WT), also known as nephroblastoma, is the most common type of kidney cancer in infants and children [1]. Along with other malignant renal tumors, WT accounts for around 7% of all childhood cancers [2, 3]. WT originates from embryonic cells during fetus development, which fail to develop properly and instead continue to grow and divide in an abnormal manner [4]. WT is characterized by disruptions in kidney embryogenesis at various stages, resulting in diverse combinations of epithelial, stromal, and blastemal cells that may even display myogenic differentiation [1]. WT typically affects only one kidney (unilateral) in most cases, but 5–10% of cases involve both kidneys (bilateral) and are more commonly seen in individuals with genetic syndromes [5]. Patients with Wilms tumor are commonly asymptomatic at the time of diagnosis, and the condition is usually identified by a parent who discovers an abdominal mass while dressing or bathing their child or by a pediatrician who palpates a mass during a routine well-child check-up. Previous reports have demonstrated that the incidence of WT varies internationally as well as with ethnicity [6–8]. The use of innovative clinical and biological prognostic factors has allowed for personalized therapy in the management of WT, resulting in significant progress in the clinical care and treatment of this disease over the last few years. The prognosis for children diagnosed with WT can vary considerably based on various factors, such as age, sex, race, chemotherapy status, laterality, and tumor [5, 9, 10]. The survival for patients with WT is strongly influenced by both their age and the stage of cancer at diagnosis, with survival rates decreasing significantly as the disease advances to higher stages (Clinic Oncol Educ) [9, 11–15]. The evolution of biological and clinical prognostic factors adopted for WT has raised the repercussions that call for assessing the [15]extent of the impact of these factors on WT. Moreover, significant differences in survival rates persist among different regions and nations, indicating the need for further efforts to reduce these disparities [16–18]. The present study aims to investigate the relevance and significance of prognostic factors previously reported in the literature, evaluate their respective impact on survival outcomes among patients with WT, and unveil the associated survival rates, relative survival, and conditional survival for a comprehensive understanding of the prognostic landscape in WT.

## Materials and methods

### Methods

We accessed the SEER*Stat Database: Incidence—SEER Research Plus Data, 17 Registries, Nov 2021 Sub (2000–2019) Using SEER*Stat software 8.3.9.2. to include patients who were diagnosed with Wilms tumor [19, 20]. We selected “Site and Morphology. Primary Site – labeled” = 'C64.9-Kidney, NOS' and “Site and Morphology. ICD-O-3 Hist/behave, malignant” = '8960/3: Nephroblastoma, NOS' based on the International Classification of Diseases for Oncology (ICDO-3) to identify patients with WT. Patients were excluded if they had an unknown SEER stage, incomplete survival data, unknown size, or unknown lymph node status.

### Operational definitions

1. SEER*Stat Database: a statistical software which calculates raw data of statistics for cancer and its rates, and trends. This software provides an intuitive and convenient mechanism, for analysis of SEER and other cancer-related databases.
2. Survival period: Survival period is defined as the difference between the time of onset of diagnosis and last follow-up or death and was reported at 1-, 5-year intervals
3. Relative survival: Relative survival serves as a comprehensive metric for gauging cancer survival exclusive of other mortality factors. It is articulated as the ratio of the observed survivors among a group of cancer patients to the expected survivors within a corresponding cohort of cancer-free individuals (https://seer.cancer.gov/seerstat/WebHelp/Relative_Survival.htm). This calculation operates under the premise of independent and competing causes of death. By accounting for the overall survival rates within the targeted population based on factors such as race, sex, age, and the date on which age was recorded, relative survival offers a normalized perspective on cancer survival outcomes.
4. Conditional survival: Conditional survival refers to the likelihood of surviving a certain timeframe (e.g., 5 years) after having already survived a specific duration following a cancer diagnosis (e.g., 1 year, 3 years, or 5 years) [21]. In instances where feasible, the calculation of conditional survival in the analysis is derived utilizing the concept of relative survival [21].
5. Standardized mortality ratios (SMR): The SMR (O/E) ratio involves the comparison of the observed deaths (O) among patients diagnosed with WT over a defined period, against the expected deaths (E) in a demographic that shares age adjustments, over the same duration.
6. Localized cancer is characterized as being limited to the organ in which it originated, without evidence of spread.
7. Regional cancer refers to the condition where the cancer has spread beyond the primary site to nearby lymph nodes or organs and tissues.
8. Distant cancer is defined as the stage at which the cancer has spread from the primary site to distant organs or distant lymph nodes. [22, 23].

### Statistical analysis

We used R software version 3.5 to calculate Kaplan–Meier estimates with 95% confidence intervals (CI) for 1- and 5-year survival. Also, we performed univariate and multivariate Cox regression models for the following factors: age, sex, race, summary stage, chemotherapy status, and laterality for Wilms cancer. We also divided age groups into < 3, > 3- < 9 years, > 9- < 15 years, and above 15 years. The computation of standardized mortality ratios (SMR) with corresponding 95% CI was performed using SEER*stat software version 8.3.9.2. The relative survival at 1, 5 and 10 years of diagnosis have been calculated with further categorization by different subgroups including age, sex and stage. Also, a five-year conditional analysis has been conducted for individuals who survived 1, 5 and 10 years after the initial diagnosis. All statistical tests were two-sided. A *P* value of less than 0.05 was considered statistically significant.

### Data collection

The SEER database was used to gather the following clinicopathological information: age at diagnosis, sex, race, laterality, lymph node status, tumor size, summary stage, and chemotherapy taking.

## Results

The study encompassed a cohort of 2273 patients. Predominantly, the study comprised females, accounting for 1219 patients (53.6%) of the total. The prevailing racial background among the participants was Caucasian, constituting 75.9% of the study population. The age distribution revealed that the most prevalent age group was < 3- > 9 years, encompassing 50.2% of the total. Tumors were localized in 980 patients, exhibited regional spread in 721 patients, and had spread to distant sites in 538 patients.

**Fig. 1:**
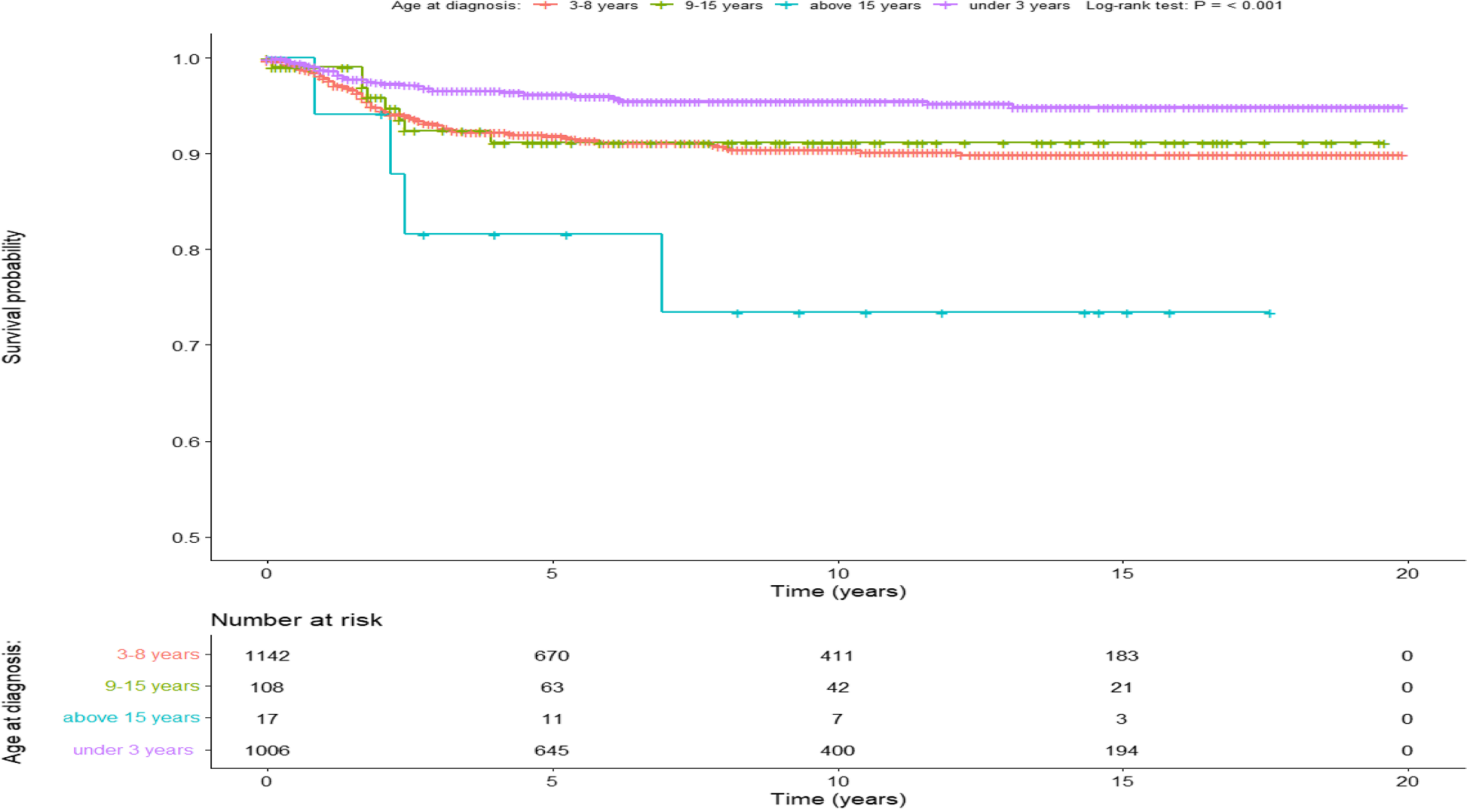
Shows a Kaplan–Meier based survival for Age variable, SEER Database

**Fig. 2:**
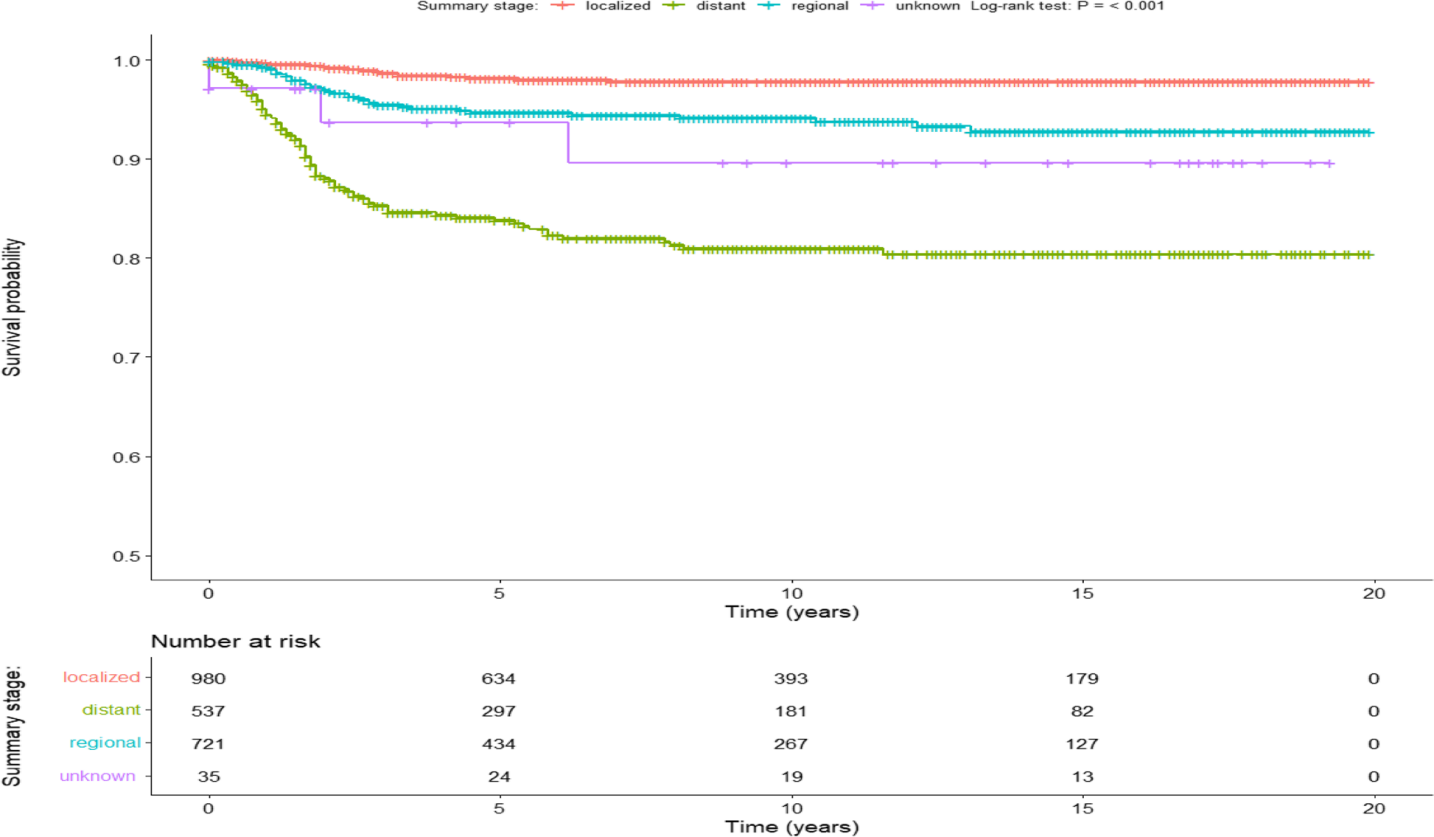
Shows a Kaplan–Meier based survival for summary stage variable, SEER Database

**Fig. 3:**
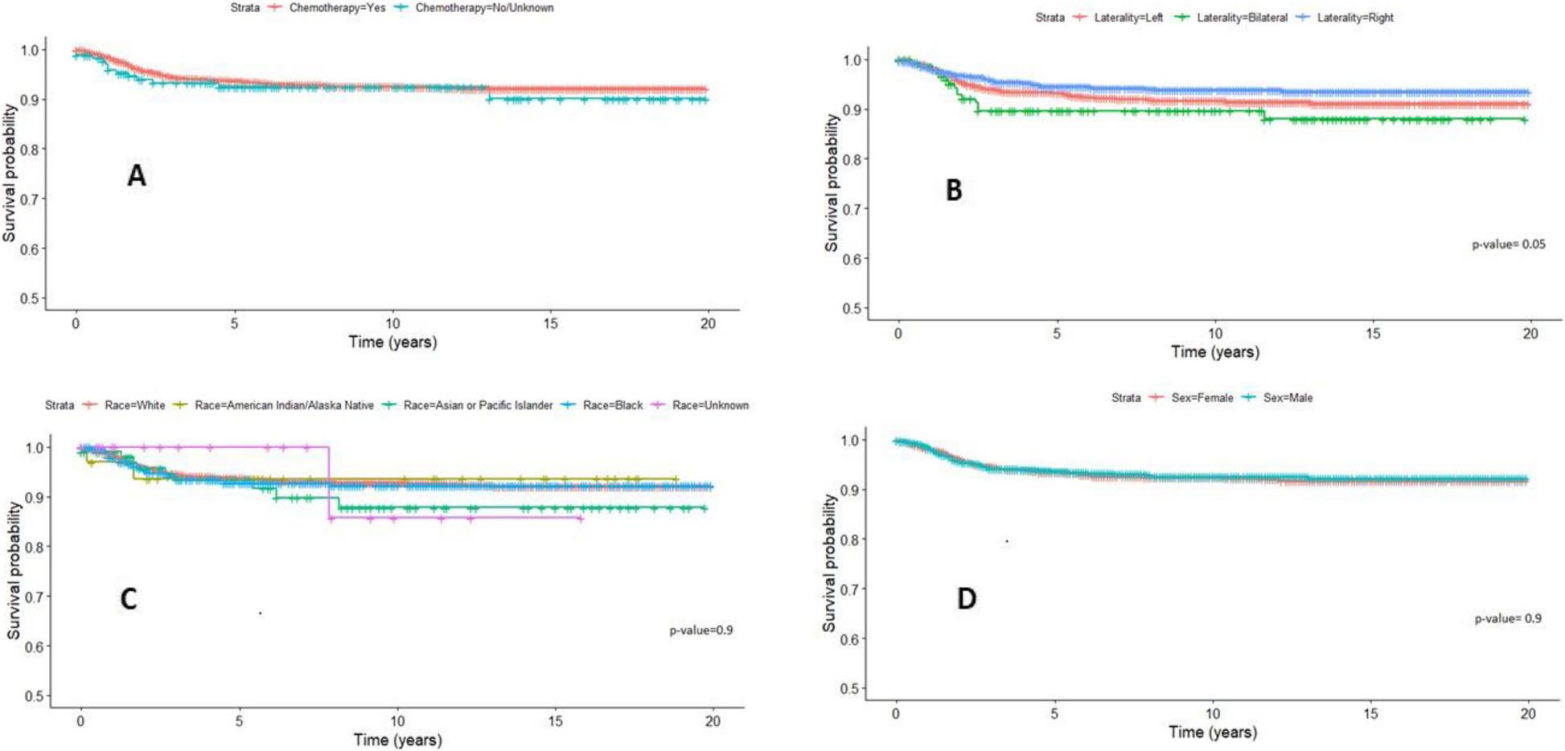
**A** Shows a Kaplan–Meier-based survival for chemotherapy variable, SEER Database. **B** Shows a Kaplan–Meier-based survival for laterality variable, SEER Database. **C** Shows a Kaplan–Meier based survival for race variable, SEER Database. **D** Shows a Kaplan–Meier based survival for sex variable, SEER Database

The overall survival (OS) rates for the entire cohort at the 5-year and 10-year marks were 93.6% (95% CI 92.6–94.7%) and 92.5% (95% CI 91.3–93.8%), respectively. Significantly poorer Cancer-Specific Survival (CSS) outcomes were observed in older age groups (log-rank p-value < 0.001). Patients aged 15 years and older exhibited a 10-year CSS of 73.4% (95% CI 53.8–100%), in contrast to patients aged 0–3 years who demonstrated a CSS of 95.5% (95% CI 94.1–97%, Fig. 1). Among patients with localized tumors, the 10-year CSS stood at 97.8% (95% CI 96.8–98.9%). However, a notable decline in CSS was observed with the increasing spread of tumors (log-rank *p* value < 0.001) (Fig. 2). Specifically, regionally spread tumors exhibited a 10-year CSS of 94.2% (95% CI 92.3–96%), while tumors with distant spread showed a 10-year CSS of 80.9% (95% CI 77.3–84.8%). Notably, factors such as chemotherapy, laterality, race, and sex did not significantly influence CSS (Table 1, Fig. 3).

**Table 1:** Survival data sub-grouped by different variables in SEER Database

Variable	Number	Survival % (95% CI) at 5 years	Survival % (95% CI) at 10 years
Overall survival	2273	93.6(2.6–94.7)	**92.5**(9**1.3**–**93.8**)
Sex			
Female	1219	93.5(92.1–95)	**92.5(90.9–94.2)**
Male	1054	93.7(92.2–95.3)	**92.6(90.8–94.3)**
Age			
< 3 years	1006	96.2(94.9–97.4)	95.5 (94.1–97)
3–8 years	1142	91.8(90.1–93.5)	90.4(99.5–92.3)
9–15 years	108	91.1(85.4–97)	91.1(85.4–97.2)
> 15 years	17	81.6(64.7–100)	73.4(53.8–100)
Race			
Asian/Pacific Islander	107	93.4(88.4–98.7)	**87.8(80.3–96.1)**
American Indian/Alaska Native	33	93.6(85.4–100)	**93.6(85.4–100)**
Black	384	92.8(90.1–95.6)	**92.2(89.3–95.3)**
White	1725	93.8(92.5–95)	**92.9(91.5–94.2)**
Unknown	24	100(100–100)	**85.7(63.3–100)**
Laterality			
Left	1085	93.3(91.7–94.9)	91.7(90–93.6)
Right	1029	94.6(93.1–96.1)	93.9(92.2–95.5)
Bilateral	159	89.7(84.7–95)	89.7(84.7–95)
Summary stage			
Distant	538	83.8(80.5–87.2)	**80.9(77.3–84.8)**
Regional	721	94.7(92.9–96.5)	**94.2(92.3–96)**
Localized	980	98.2(97.2–99)	**97.8(96.8–98.9)**
Unknown	35	93.7(85.5–100)	**89.6(79.1–100)**
Chemotherapy			
Yes	2091	93.7(92.6–94.8)	92.5(91.3–93.8)
No	182	92.5(88.5–96.7)	92.5(88.5–96.7)

Univariate Cox proportional hazard regression analyses were undertaken to identify significant prognostic factors for CSS in patients with Wilm’s tumor. Patients below the age of 3 demonstrated notably superior CSS compared to those aged 3–8 (crude hazard ratio (cHR) 0.48, 95% CI 0.33–0.69, *p* < 0.001). Conversely, CSS was significantly poorer in patients older than 15 years compared to those aged 3–8 years (cHR 2.72, 95% CI 1–7.42, *p* = 0.049). In comparison to patients with localized tumors, those with distant spread exhibited significantly worse CSS with a cHR of 10.24 (95% CI 6.09–17.23, *p* < 0.001). Similarly, patients with regional spread experienced a significantly lower CSS, with a cHR of 3.09 (95% CI 1.74–5.47, *p* < 0.001), as did those with an unknown stage, with a cHR of 4.97 (95% CI 1.46–16.95, *p* = 0.01).

Laterality, sex, chemotherapy, and race were found to have no significant impact on CSS as detailed in Table 2. All factors identified as significant in the univariate Cox regression were included in the multivariate model. Surprisingly, age did not emerge as a significant predictor of CSS. However, patients with distant spread had a markedly worse CSS (adjusted hazard ratio (aHR) 9.22, 95% CI 5.42–15.69, *p* < 0.001), as did those with regional spread (aHR 2.84, 95% CI 1.6–5.07, *p* < 0.001), and individuals with an unknown stage (aHR 4.98, 95% CI 1.46–17.01, *p* = 0.01) when compared to patients with localized tumors (Table 2).

**Table 2:** Univariate and multivariate cox regression models for the different factors

	**Univariate Regression**			**Multivariate Regression**		
	**HR**^**a**^	**95% CI**	***P* value**	**HR**^**a**^	**95% CI**	***P* value**
**Age (vs 3–8 years)**						
** < 3**	**0.48**	**(0.33–0.69)**	** < 0.001**	**0.72**	**(0.49–1.05)**	**0.08**
**9–15**	**0.92**	**(0.45–1.90)**	**0.83**	**0.86**	**(0.42–1.77)**	**0.68**
** > 15**	**2.72**	**(1–7.42)**	**0.049**	**2.59**	**(0.95–7.05)**	**0.06**
**Summary Stage (vs Localized)**						
**Distant**	**10.24**	**(6.09–17.23)**	** < 0.001**	**9.22**	**(5.42–15.69)**	** < 0.001**
**Regional**	**3.09**	**(1.74–5.47)**	** < 0.001**	**2.84**	**(1.6–5.07)**	**0. < 0.001**
**Unknown**	**4.97**	**(1.46–16.95)**	**0.01**	**4.98**	**(1.46–17.01)**	**0.01**
**Laterality (vs Left)**						
**Right**	**0.73**	**(0.52–1.04)**	**0.08**			
**Bilateral**	**1.41**	**(0.81–2.45)**	**0.23**			
**Sex (vs Female)**						
**Male**	**0.96**	**(0.7–1.34)**	**0.83**			
**Chemotherapy (vs Yes)**						
**NO**	***1.18***	***(0.67–2.09)***	***0.57***			
**Race (vs White)**						
**American Indian/Alaska Native**	**0.95**	**(0.23–3.85)**	**0.94**			
**Asian/Pacific Islander**	**1.42**	**(0.72–2.8)**	**0.31**			
**Black**	**1.06**	**(0.68–1.63)**	**0.80**			
**Unknown**	**0.85**	**(0.12–6.08)**	**0.87**			

^a^This number represents the hazard ratio for Cancer-specific causes for the above co-variables. All statistical tests were two-sided

### Causes of death in Wilms tumor patients

Among patients diagnosed with WT, the foremost cause of death was attributed to renal cancers [140; 77.3%, SMR = 10,396.70 (8745.9– 12,268.51)], followed by non-renal cancers [20; 11%, SMR = 43.07 (26.31– 66.52)], and non-cancer causes [20; 11%, SMR = 2.21 (1.35–3.41). However, it is noteworthy to mention that The SMR for non-cancer causes showed no significance in the initial 2 years but demonstrated a significant increase in subsequent years.

### Relative survival and conditional survival analysis

The one-year, five-year, and ten-year relative survival for WT patients were 98.10%, 95%CI (97.50–98.60), 92.80%, 95%CI (91.60–93.90) and 91.3%, 95%CI (89.9–92.5) respectively. The five-year conditional survival after one year, five years and ten years of survival for WT were 94.0%%, 95%CI (92.9–95.0), 98.4%, 95%CI (97.5–98.9) and 99.0%, 95%CI (97.7–99.6) respectively. This means that the longer a person has already survived after diagnosis with cancer, the greater the likelihood that person will survive their cancer for another 5 years or more. Additional results regarding survival rates categorized by different subgroups, including age < 3, > 3- < 9 years, > 9- < 15 years, and above 15 years, and gender (male and female), can be found in Tables 3 and 4.

**Table 3:** Relative Survival and Conditional Survival Analysis among Wilms cancer patients subgrouping by age

Survival	N	Relative Survival	Relative cumulative CIs lower	Relative cumulative CIs upper
Cumulative summary/ < 3				
12 months	**1,080**	**98.20%**	**97.20%**	**98.90%**
60 months	**1,080**	**95.1%**^**c**^	**93.5%**^**c**^	**96.4%**^**c**^
120 months	**1,080**	**93.9%**^**c**^	**92.0%**^**c**^	**95.3%**^**c**^
5 yrs conditional at 1 year	**985**	**96.4%**^**c**^	**94.9%**^**c**^	**97.5%**^**c**^
5 yrs conditional at 5 year	**701**	**98.7%**^**c**^	**97.3%**^**c**^	**99.4%**^**c**^
5 yrs conditional at 10 years	**442**	**99.1%**^**c**^	**96.7%**^**c,a**^	**99.7%**^**c,a**^
Cumulative summary/ > 15				
12 months	**19**	**94.8%**^**c**^	**67.9%**^**c,a**^	**99.3%**^**c,a**^
60 months	**19**	**83.7%**^**c**^	**57.2%**^**c**^	**94.5%**^**c**^
120 months	**19**	**76.3%**^**c**^	**47.2%**^**c**^	**90.7%**^**c**^
5 yrs conditional at 1 year	**18**	**88.3%**^**c**^	**60.5%**^**c**^	**97.0%**^**c**^
5 yrs conditional at 5 year	**13**	**91.0%**^**c**^	**50.3%**^**c,a**^	**98.7%**^**c,a**^
5 yrs conditional at 10 years	**7**	**100.0%**^**b,c**^	^**c,d**^	^**c,d**^
Cumulative summary/3–8 yrs				
12 months	**1,221**	**98.00%**	**97.10%**	**98.70%**
60 months	**1,221**	**91.00%**	**89.10%**	**92.60%**
120 months	**1,221**	**89.5%**^**c**^	**87.3%**^**c**^	**91.3%**^**c**^
5 yrs conditional at 1 year	**1,109**	**92.2%**^**c**^	**90.3%**^**c**^	**93.7%**^**c**^
5 yrs conditional at 5 year	**723**	**98.3%**^**c**^	**96.9%**^**c**^	**99.1%**^**c**^
5 yrs conditional at 10 years	**451**	**98.7%**^**c**^	**96.6%**^**c**^	**99.5%**^**c**^
Cumulative summary/9–15 yrs				
12 months	**119**	**98.3%**^**c**^	**93.3%**^**c,a**^	**99.6%**^**c,a**^
60 months	**119**	**91.3%**^**c**^	**83.8%**^**c**^	**95.4%**^**c**^
120 months	**119**	**88.5%**^**c**^	**79.6%c**	**93.7%**^**c**^
5 yrs conditional at 1 year	**106**	**91.6%**^**c**^	**83.7%**^**c**^	**95.8%**^**c**^
5 yrs conditional at 5 year	**73**	**97.0%**^**c**^	**87.0%**^**c,a**^	**99.3%**^**c,a**^
5 yrs conditional at 10 years	**49**	**100.0%**^**b,c**^	^**c,d**^	^**c.d**^
Cumulative summary/overall				
12 months	**2,439**	**98.10%**	**97.50%**	**98.60%**
60 months	**2,439**	**92.80%**	**91.60%**	**93.90%**
120 months	**2,439**	**91.3%**^**c**^	**89.9%**^**c**^	**92.5%**^**c**^
5 yrs conditional at 1 year	**2,218**	**94.0%**^**c**^	**92.9%**^**c**^	**95.0%**^**c**^
5 yrs conditional at 5 year	**1,510**	**98.4%**^**c**^	**97.5%**^**c**^	**98.9%**^**c**^
5 yrs conditional at 10 years	**949**	**99.0%**^**c**^	**97.7%**^**c**^	**99.6%**^**c**^

Actuarial method. Ederer II method used for cumulative expected

Confidence interval: Log(-Log()) Transformation. The level is 95%

^a^The width of the confidence interval is more than 25% larger than if the normal approximation was applied

^b^The relative cumulative survival is over 100 percent and has been adjusted

^c^The relative cumulative survival increased from a prior interval and has been adjusted

^d^ The statistic could not be calculated

**Table 4:** Relative Survival and Conditional Survival Analysis among Wilms cancer patient’s subgrouping by sex

Survival	N	Relative survival	Relative cumulative cis lower	Relative cumulative cis upper
Cumulative summary/male				
12 months	**1,129**	**98.40%**	**97.40%**	**99.00%**
60 months	**1,129**	**92.80%**	**90.90%**	**94.20%**
120 months	**1,129**	**91.1%**^**a**^	**89.1%**^**a**^	**92.9%**^**a**^
5 yrs conditional at 1 year	**1,041**	**93.7%**^**a**^	**92.0%**^**a**^	**95.1%**^**a**^
5 yrs conditional at 5 year	**714**	**98.3%**^**a**^	**96.8%**^**a**^	**99.1%**^**a**^
5 yrs conditional at 10 years	**453**	**98.8%**^**a**^	**96.3%**^**a**^	**99.6%**^**a**^
Cumulative summary/female				
12 months	**1,310**	**97.90%**	**96.90%**	**98.60%**
60 months	**1,310**	**92.9%**^**a**^	**91.2%**^**a**^	**94.3%**^**a**^
120 months	**1,310**	**91.4%**^**a**^	**89.5%**^**a**^	**93.0%**^**a**^
5 yrs conditional at 1 year	**1,177**	**94.3%**^**a**^	**92.6%**^**a**^	**95.5%**^**a**^
5 yrs conditional at 5 year	**796**	**98.4%**^**a**^	**97.1%**^**a**^	**99.1%**^**a**^
5 yrs conditional at 10 years	**496**	**99.1%**^**a**^	**97.6%**^**a**^	**99.7%**^**a**^

Actuarial method. Ederer II method used for cumulative expected

Confidence interval: Log(-Log()) Transformation. The level is 95%

^a^The relative cumulative survival increased from a prior interval and has been adjusted

## Discussion

### The main findings of our study

The younger age group predominated in our study with better outcomes. Locally spreading tumors have the best survival. Renal Cancer was the most common cause of death.

### Analysis of the findings

A total of 2273 patients were included in our study; 53.6% of patients were females. The most common race was Caucasian (75.9%), and the most common age group was > 3- < 9 years (50.2%). The tumors were localized in 980 patients, had spread regionally in 721 patients, and had spread to distant sites in 538 patients. 5-year and 10-year OS for the entire cohort were 93.6% and 92.5%, respectively. Age was not a significant predictor of CSS. Patients with distant spread, regional spread, and those with unknown stage had worse CSS than those with localized tumors. According to our study, majority of the patients were female (53.6%). The percentage of female patients is comparable to those in previous studies; for example, a study conducted in 2014–2016 was 52% [24]; conducted in 1988–2010, it was 52.1% [25]. Likewise, male patients were 47.46% in another study conducted from 2004 to 2018 [26]. Male sex was the sole significant factor, where males have a lower hazard ratio [27]. But it is contradicted in a study conducted in 2006–2010 as the male-to-female ratio was 1.55:1 [28]. Also, a study conducted in 2000 -2021 showed fatal outcomes for male sex [29]. Another study was done recently in 2020, where PDL 1 ligands were studied, and the levels were higher in females, and it was associated with bad outcomes. These results support our study that females have more prevalence and worse outcome when compared to male patients [30].

Recent research in 2020 described the fact that the most important prognostic factor is the histological subtype of the tumor. This study showed that the survival was 100% for boys and 76.8% ± 1.6 for girls. So, sex has been an independent prognostic factor in determining the survival of children with Wilms tumor [31]. In our study, laterality, sex, chemotherapy, and race did not affect the outcome of the Wilms tumor. However, our results disagreed with a previous study done in 2016, according to which laterality of the tumor and sex has been affecting the outcome of the Wilms tumor along with the histological subtype and stage at presentation [32]. However, the late presentation can also affect the survival of WT, and recent research was done in 2019 to investigate the causes of this delayed presentation. Poor maternal education and inappropriate antenatal care were found to be associated with late presentation and hence less survival rates in WT [33].

In our study, average age group is > 3- < 9 years (50.2%). In the previously mentioned study, the average age was three years and two months, and the percentage of less than six years old patients was 88% [24, 25].

Undoubtedly, judging a patient's prognosis based on just a single variable may cause deviation. So, including multiple prognostic factors has always been the best approach towards this [25]. In our study, we include CSS as the prognostic factor as patients with distant spread, regional spread, and those with unknown stage had worse CSS when compared to patients with localized tumors. A total of 43.5% of patients had localized, 32.3% had regionally spread, and 24.02% had distant metastasis of tumor. 53.85% metastasis was noted in a study conducted in 2014–2016 [24]. It was stated to be 41.63% local, 36.27% regional, and 22.1% distant metastatic in another study which is very much comparable to the values found in our study [26]. It was found to be 45.3% local, 31.2% regional, and 23.5% metastatic in another study [25]. Metastatic disease has a poor outcome. Though therapeutic improvement has been made in the treatment of WT over the past decade, there is still a lot to be done to improve the outcome of patients with metastatic [34].

In our study, CSS is worse for patients older than 15 years of age, 73.4% for ten years old, and 95.5% for 0–3 years old patients. In a study conducted in 1988 -2010, it is 79% for three years old and 76% for five years old patient [25]. These results also showed a better outcome in young patients, as proved by our study.

Geographic and socioeconomic factors are still considered to have direct relationships with the prognosis of several diseases. When adopting contemporary pediatric oncology cooperative group methods, children with Wilms tumors have an overall survival rate of approximately 90% in countries with high incomes [35, 36]. However, whereas patient outcomes in high-income countries are outstanding, patient outcomes in low- and lower-middle-income countries are not as good, with survival rates of fewer than 50% [37, 38]. In low- and lower-middle-income countries, treatment abandonment, delayed diagnosis, delayed surgery, advanced disease at presentation, metastatic disease at diagnosis, unfavorable histology, larger tumor volume, malnourishment, recurrence of the disease, and subpar treatment are among the known poor prognostic factors [38–40]. Since several of these factors are modifiable its curcial to increase the effrots to overcome these challenges through the implementation of appropriate strategies.

However, some low- and lower-middle-income countries such as Eygpt has recorced a great progress despite all the mentioned obstacles. In 2020, a study conducted by Asfour et al., aimed to assess the clinical outcome and the different prognostic factors that influence the outcome of pediatric loco-regional WT cases treated at National Cancer Institute, Cairo University, Egypt. According to the results obtained from this study, Egypt had OS nearly the same as in developed countries [41].

Survival rates for children diagnosed with a WT are subject to diverse factors. These factors encompass the tumor's stage, the individual's age and overall health, as well as the efficacy of the treatment plan. The available data regarding WT survival rates in the literature is limited. The reported 5-year relative survival rate for children with a WT by the American Cancer Society is 93% [42]. They also reported that the risk of WT to come back after treatment is between 15 and 50%, and it is most likely to come back within the first 2 years following treatment [42]. Another study from Uganda reported that the one-year overall survival of WT was found to be 59.3% (95% CI: 40.7–73.3) [39]. Survival rates for included WT patients in this study showcase a strong trend, with impressive one-year (98.10%), five-year (92.80%), and ten-year (91.3%) relative survival rates. Also, the corresponding five-year conditional survival rates after 1 year, five years, and ten years are equally promising at 94.0%, 98.4%, and 99.0%, respectively. These findings underscore the encouraging prospect that the longer a person has successfully battled cancer, the more favorable their chances of extending their survival for an additional 5 years or more.

### Future perspectives

Further studies examining the prognosis through longer periods may be needed to address other possible prognostic factors.

### Strength of the study

Our study included a sample size of 2273 which was a pretty much large sample than most of the studies, so it adds to the strength of our study. It is based on a considerable period (19 years of study), making the result more generalized and reliable.

## Conclusion

Management and follow-up should be tailored to the specific needs for each WT patient. Our findings provide an insightful way for monitoring future risk factors among WT patients. Staging has a significant impact on survival outcome.

## Data Availability

The datasets generated and analyzed during the current study are available in the SEER*Stat Database: Incidence—SEER Research Plus Data, 17 Registries, Using SEER*Stat software 8.4.0.1 http://www.seer.cancer.gov/seerstat repository.

## Abbreviations

aHR: adjusted hazard ratio
cHR: crude hazard ratio
CSS: Cancer-Specific Survival
ICDO-3: International Classification of Diseases for Oncology
NOS: Nephroblastoma
OS: Overall survival
SEER: The Surveillance, Epidemiology, and End Results
WT: Wilms tumor
